# Targeting self-efficacy more important than dysfunctional behavioral cognitions in patients with longstanding chronic low back pain; a longitudinal study

**DOI:** 10.1186/s12891-021-04637-3

**Published:** 2021-09-25

**Authors:** Miranda L. van Hooff, Johanna E. Vriezekolk, Robert Jan Kroeze, John K. O’Dowd, Jacques van Limbeek, Maarten Spruit

**Affiliations:** 1 Department of Research, Sint Maartenskliniek, P.O. Box 9011, 6500 GM Nijmegen, The Netherlands; 2 Department of Orthopedics, Radboud University Medical Center, P.O. Box 9101, 6500 HB Nijmegen, The Netherlands; 3 Department of Orthopedics, Sint Maartenskliniek, P.O. Box 9011, 6500 GM Nijmegen, The Netherlands; 4 The RealHealth Institute, 23-31 Beavor Lane, London, W6 9AR UK; 5 Independent Epidemiologist, Nijmegen, The Netherlands

**Keywords:** Low back pain, Cohort studies, Pain management, Disability, Behavior, Self efficacy, Catastrophization, Self-management, Spine

## Abstract

**Background:**

Multidisciplinary pain management programs based on cognitive behavioral training (CBT) principles have been shown moderately effective in improving daily functioning in patients with chronic low back pain (CLBP). To optimize health-related outcomes as daily functioning, a clear understanding of the working mechanisms of these programs is warranted. Being confident to achieve a desired outcome, i.e. self-efficacy, is suggested to be a more potent determinant for beneficial treatment outcomes than restructuring the patient’s dysfunctional behavioral cognitions (pain catastrophizing and fear of movement [FoM]), but the evidence is scarce. The objective of this study was twofold: 1) to determine whether a two-week pain management program resulted in post-treatment improvements in self-efficacy and decreased dysfunctional behavioral cognitions in patients with CLBP, and 2) to examine the unique contribution of self-efficacy to improvement in post-treatment disability.

**Methods:**

A secondary analysis of an historical cohort study was performed, including 524 patients (59% females). Primary outcome: functional status (Oswestry Disability Index v2.1a). Secondary outcomes: catastrophizing (Pain Catastrophizing Scale), FoM (Tampa Scale for Kinesiophobia), and self-efficacy (Pain Self-Efficacy Questionnaire). Assessments: pre-, post-treatment, 1, and 12-months follow-up. Paired Student’s t-tests were applied and clinical relevancy of improvements was described using minimal clinical important changes. Adjusted multivariate linear regression analyses were performed to explore the unique contribution of self-efficacy.

**Results:**

The mean age of patients was 46 (SD = 9.5) years and they had longstanding CLBP (mean 12.5 [SD = 10.8] years). Mean functional status, self-efficacy, and dysfunctional behavioral cognitions improved significantly at post-treatment, with improvements maintained at 12 months follow-up. Post-treatment relevant improvements in self-efficacy and dysfunctional behavioral cognitions ranged from 62.4% (FoM) to 68.7% (self-efficacy). Post-treatment self-efficacy improved the model explaining post-treatment functional disability (basic model *R*^2^ = 0.49, F_(6,517)_ = 83.67, *p* < 0.001; final model *R*^2^ = 0.57, F_(8,515)_ = 85.20, p < 0.001). This was further substantiated by the relative contribution (standardized betas) of self-efficacy: 5.67 times more than catastrophizing and 9.75 times more than FoM.

**Conclusions:**

Targeting self-efficacy contributes to fast improvement in functional status for selected and motivated patients with persistent CLBP. In pain management programs and (online) self-management programs for CLBP, targeting patients’ self-efficacy should have a prominent place.

## Introduction

Worldwide low back pain (LBP) is a highly common health condition, affecting all age groups, and getting more prevalent with the increasing and ageing world population [1, 2]. In a series of papers of The Lancet LBP Series Working Group the authors called for action to develop and promote multidisciplinary and patient-centred care focusing on self-management and healthy lifestyles as a means of restoring and maintaining daily functioning and optimizing (social) participation [3]. Multidisciplinary bio-psychosocial programs, based on cognitive behavioural training (CBT) principles targeting dysfunctional behavioural cognitions (catastrophizing, fear of movement), have shown to be effective in improving daily functioning in CLBP-patients [4, 5]. Although recommended in international guidelines [6–8], the working mechanisms are not yet fully understood.

To gain insight in the working mechanisms we previously evaluated a two-week, highly intense, residential combined physical and psychological (CPP) pain management program for patients with longstanding severe CLBP. Post-treatment clinically relevant improvements were shown in self-efficacy beliefs and disability, resulting to ‘normal’ healthy functioning that remained stable after a minimum of five-year follow-up [9, 10]. Although it is assumed that in patients with longstanding persistent pain, dysfunctional behavioural cognitions are resistant to change [11], the fast improvements implicate that these dysfunctional behavioural cognitions can be changed. It is still unclear to what extent these changes are responsible for improvements in functional outcomes. That would provide evidence that these indicators are important treatment targets, and part of the working mechanisms.

Furthermore, the question whether (pain) self-efficacy, i.e. confidence and belief in one’s ability to achieve a desired outcome [12], uniquely contributes to improved functional outcome in longstanding CLBP, is still unresolved. Examining whether targeting self-efficacy and to what extent changes in self-efficacy uniquely contribute to improvement in functional outcome is important to understand the working mechanisms. In a systematic review of Jackson et al. [13], including 15,616 chronic pain patients (83 studies), the relevance is shown as it facilitates recovery in terms of lower levels of disability and pain. CLBP programs based on CBT principles are likely to improve self-efficacy, leading to improved disability and pain [2, 14, 15]. As such, pain self-efficacy could be a more important determinant than fear of movement in explaining disability [16, 17].

The purpose of this longitudinal study with CLBP-patients was twofold: 1) to determine whether a two-week residential CPP pain management program resulted in post-treatment improvements in self-efficacy and decreased dysfunctional behavioural cognitions and subsequently, 2) to examine the unique contribution of self-efficacy to improvement in post-treatment disability. We hypothesized that the CPP pain management program is able to decrease both pain catastrophizing and fear of movement, due to post-treatment improvements in self-efficacy, and that these improvements are maintained after a year.

## Methods

### Study design

The current cohort study is a secondary analysis with data obtained for a previously published cohort study that follows the STROBE guidelines [18]. The hospital’s review board approved the study protocol. Ethical approval for this study was not deemed required, as the Dutch Act on Medical Research involving Human Subjects does not apply to screening questionnaires that are part of routine clinical practice. All patients were informed about the study and gave informed consent. For this study, fully anonymized data were used.

### Participants

The orthopaedic spine surgeons consecutively recruited the study participants. Patients were referred to the two week residential CBT-based CPP pain management program if they met the following main inclusion criteria: age between 18 and 65 years, low back pain for at least six months, failed conservative primary care treatment and no indication for surgical or other invasive pain treatment confirmed by spinal surgeons at the orthopaedic outpatient department, able to speak and read Dutch, expressed their willingness and motivation to behavioural change, and wished to be enrolled in the intensive two-week residential treatment. The main exclusion criteria were diagnosed psychiatric disorders and involvement in litigation claims. Final enrolment was based on an extensive screening by the multidisciplinary team consisting of a psychologist, a physiotherapist, an occupational therapist, and a psychomotor therapist. During the screening patients’ willingness to participate and motivation for behavioral change are discussed and the exclusion criteria checked (e.g. to ascertain whether patients’ mental and/or physical problems would interfere with the program).

### Procedure

#### Intervention

A detailed description of the bio-psychosocial CPP pain management program has previously been reported [19]. This program is based on CBT and exposure principles and is designed as a so-called ‘pressure cooker’ modelled program (100-h principle). Briefly, the two-week program is delivered in a hotel facility by a multidisciplinary team of allied healthcare professionals in collaboration with and under the supervision of the orthopaedic spine surgeons. The team has been trained in CBT and exposure techniques for chronic pain. The program comprises an intake assessment day, the 10-day residential program and two days for the post-treatment follow-up assessment at one and 12 months. The intervention involves approximately 100 h of participant contact time delivered in a group-orientated residential setting, including intake assessment and follow-up assessments. The main aim of the intervention is to improve daily functioning. This goal is achieved by increasing the participant’s capability for self-management (self-efficacy), addressing the psychological impact of pain and dysfunctional behavioural cognitions (catastrophizing and fear of movement), and increasing physical condition; all directed toward enhancing return to work. The program is described in a manual and consists of a range of CBT and exposure techniques and self-management principles, such as patient education, cognitive restructuring, exposure to fearful situations, relaxation techniques, pacing, goal setting, and homework assignments. Physical training is an integral part of the treatment and includes daily exercises to improve muscle strength, range of movement, and physical fitness.

#### Measurement and data collection

As part of routine outcome assessment procedures, participants completed questionnaires at intake (pre-treatment), post-treatment (at the end of the two-week program), at one and at 12 months post-treatment. Furthermore, at pre-treatment participants provided information on medical history including previous surgery, pain history, current pain intensity (Numeric Rating Scale [NRS 0–100]) [19], pain medication use, and employment status. At each assessment participants completed questionnaires on functional status, pain severity, pain self-efficacy, pain catastrophizing and fear of movement. All instruments used had previously been validated in Dutch CLBP samples.

### Primary outcome measure

#### Functional status

The Dutch version of the Oswestry Disability Index (ODI v2.1a) [20, 21] was used to assess functional status. The 10-item ODI measures the impact of LBP on daily functioning in ten domains of daily life. The total ODI score is computed by summing the item scores; ranging from 0 to 100, with higher scores indicating greater disability. A relative change measure, minimal clinical important change (MCIC) of 10 points was used to indicate relevant improvement [22, 23].

### Secondary self-report outcome measures

#### Self-efficacy

The Pain Self-Efficacy Questionnaire (PSEQ) was used to assess participants’ ability to self-manage his or her back pain complaints. The Pain Self-Efficacy Questionnaire (PSEQ) is a 10-item inventory that measures patient’s belief about his/her ability to accomplish a range of activities despite his/her pain [24]. Scores range from 0 to 60, with higher scores indicating stronger self-efficacy beliefs. An MCIC of 5.5 points was used to indicate relevant improvement [25].

#### Catastrophizing

The Dutch version of the Pain Catastrophizing Scale (PCS) was used to assess pain-related catastrophizing [26, 27] was used to indicate change in catastrophizing. The 13 items can be divided into three subscales (rumination, magnification and helplessness) and are scored on a 5-point ordinal scale. The total score ranges between 0 and 52 points, with higher scores indicating higher levels of pain catastrophizing. An MCIC of 5.8 points was used to indicate relevant improvement [28].

#### Fear of movement

The Tampa Scale for Kinesiophobia (TSK) was used to assess fear of movement/(re-)injury in individuals with pain [29, 30]. The 17 items are scored on a 4-point ordinal scale. The total score ranges between 17 and 68 points, with higher scores indicating higher levels of fear of movement. An MCIC of 4.5 points was used to indicate relevant improvement [28].

### Data analyses

Data were checked and found normally distributed using the Shapiro-Wilk test. Descriptive patient characteristics at pre-treatment were summarized. Categorical data were described in counts and percentages, whereas continuous variables were described as means and standard deviations (SD). To indicate whether there are relevant differences between complete and incomplete cases that might hamper the generalization of findings, the percentage of ‘partial cases’ (i.e. participants who did not respond to questionnaires) was calculated, and the pre-treatment characteristics of partial cases and complete cases were compared with Pearson chi-square tests for categorical variables and independent Student’s *t* tests for continuous variables.

### Missing data

To account for missing data on primary and secondary outcome measures (partial cases) and to avoid possible bias by excluding the data of these participants, a multiple imputation (MI) technique (*n* = 10 sets) was performed. To generate the values to impute an augmentation Markov chain Monte Carlo replacement method was used under the assumption that the data were ‘missing at random’.

### First hypothesis: improvement in dysfunctional behavioural cognitions and self-efficacy

A paired Students’ *t*-test was used to examine pre- and post-treatment values and post-treatment and 1 year follow-up assessment values. Percentages of patients reaching MCIC at post-treatment were described and effect sizes (Cohens’ *d*) were calculated to indicate the magnitude of changes during the two-week program. Cohen’s *d* is defined as the difference between means of the pre-treatment assessment and the post-treatment assessment, divided by the pooled standard deviation (SD). An effect size of 1 is approximately equivalent to a change of 1 SD in the sample: an effect size (*d*) of 0.2 is considered small, 0.5 moderate, while 0.8 indicates a large effect [31]. Partial *R*^2^ was computed using GLM repeated measures ANOVA to estimate the relevance of the treatment effect.

### Second hypothesis: unique contribution of self-efficacy to post-treatment disability

Multivariate linear regression techniques were applied. Linear models were built to examine the influence of self-efficacy, pain catastrophizing, and fear of movement on the post-treatment functional status. Before constructing the models, the models’ assumptions were checked [32]: linear relationships (Levene’s test), no perfect multicollinearity (Pearson’s [*r*] < 0.80; Variance Inflation Factors < 10, and the corresponding tolerance levels > 0.2), homoscedasticity between secondary (independent) outcome indicators and primary (dependent) outcome indicators, and the balance between the number of predictive indicators and the number of outcome events was considered, which is recommended not to be lower than 10–15 events per factor (EPV) [33, 34].

Model development.

Post-treatment functional status was used as a continuous (dependent) outcome indicator. In one block, the pre-treatment values on primary and both pre and post-treatment values on secondary outcomes, to indicate change, were entered into the model as independent variables. To adjust for pre-treatment differences in experienced pain, the baseline pain intensity score was added. To calculate the relative contribution of post-treatment self-efficacy to other independent variables, standardized beta coefficients (β) were used. The models’ performances were assessed by the percentage of variance explained (i.e. Nagelkerke’s *R*^*2*^).

Data analyses were performed using STATA (version 13.0 for Windows; StataCorp, College Station, Texas, USA). The level for statistical significance was set at *p* < 0.05.

## Results

### Response

In total 727 CLBP-patients were referred to the program. After the multidisciplinary intake 524 (72.1%) were included and participated in the program. Of this sample (*n* = 524), 67 participants (12.8%) had missing data at least at one post-treatment assessment after the pre-treatment assessment [19]. At pre-treatment assessment, the characteristics and the pre-treatment values on measurement instruments were not significantly different from participants with complete data.

### Pre-treatment characteristics (Table 1)

The mean age of the total study population was 45.4 (SD = 9.6) years and the majority was female (58%). On average, the participants had longstanding CLBP complaints (mean 12.5 [SD = 10.8] years). The majority of the participants were at work (68%) at the pre-treatment assessment and one–third had previously undergone surgery for their LBP (32%).

**Table 1:** Pre-treatment characteristics of the total study sample, complete, and partial cases

Pre-treatment characteristics	Total sample (*n* = 524)	Complete cases (*n* = 457)	Partial cases (*n* = 67)
*Sociodemographic*			
Age, mean (SD) in years	45.4 (± 9.6)	45.5 (± 9.6)	44.5 (± 10.3)
Gender, Female n (%),	303 (57.8)	270 (59.1)	33 (49.3)
Employment status, Yes n (%)	356 (67.9)	317 (69.4)	39 (58.2)
*CLBP History*			
Duration of LBP, mean (SD) in years	12.5 (±10.8)	12.4 (±10.8)	13.4 (±10.7)
Pain medication, Yes n (%)	454 (86.6)	397 (86.9)	57 (85.1)
Previous surgery, Yes n (%)	169 (32.3)	142 (31.1)	27 (40.3)
Current pain intensity (NPRS), mean (SD)	60.7 (± 21.1)	32.5 (± 10.8)	31.2 (± 12.1)
*Primary outcome*			
Functional status (ODI), mean (SD)	41.4 (± 14.1)	41.1 (± 14.1)	43.9 (± 15.8)
*Secondary outcomes*			
Self-efficacy (PSEQ), mean (SD)	32.4 (± 10.8)	32.5 (± 10.8)	31.2 (± 12.1)
*Dysfunctional behavioural cognitions*			
Catastrophizing (PCS), mean (SD)	22.9 (± 8.9)	22.9 (± 8.8)	24.0 (± 9.5)
Fear of movement (TSK), mean (SD)	39.6 (± 6.4)	39.6 (± 6.4)	40.7 (± 6.7)

*ODI* oswestry disability index; *PSEQ* pain self-efficacy questionnaire; *PCS* pain catastrophizing scale; *TSK* tampa scale for kinesiophobia; *NPRS* numeric pain rating scale

#### Improvement in dysfunctional behavioural cognitions and self-efficacy (Table 2)

Mean primary and secondary outcome scores improved significantly between pre- and post-treatment assessments. In two third of the patients a relevant improvement in self-efficacy was shown after two weeks (MCIC 68.7%). The percentage patients reaching MCIC for dysfunctional behavioural cognitions ranged from 62.0% (catastrophizing) to 62.4% (fear of movement). Large effect sizes were found for self-efficacy (*d* = 1.06, *R*^2^ = 0.53) and the dysfunctional behavioural cognitions (i.e. catastrophizing: *d* = 1.08, *R*^2^ = 0.52; fear of movement: *d* = 1.06, *R*^2^ = 0.50); a moderate effect size was found for functional disability (*d* = 0.59, *R*^2^ = 0.23). Between the post-treatment and the one-year follow-up assessment, a slight statistically significant improvement was observed for functional disability (t_(1,523)_ = 12.37, *p* < 0.001), catastrophizing (t_(1,523)_ = 23.73, p < 0.001) and fear of movement (t_(1,523)_ = 23.01, p < 0.001). Self-efficacy remained stable (t_(1,523)_ = 0.66, *p* = 0.51). Moreover, for all outcomes no relevant 1-year follow-up time effect is seen (*R*^*2*^ ranging from 0.001 to 0.21), indicating that the improvements seen directly post-treatment were maintained in the first year following the treatment.

**Table 2:** Mean (SD), paired t-test values, and Cohens’ *d* effect sizes (n = 524)

	Pre-treatment	Post-treatment	1 yr FU	Pre versus Post			Post versus 1 yr FU	
				***t***_**(1,523)**_	***R***^***2***^	***d***	***t***_**(1,523)**_	***R***^***2***^
**Primary outcome**								
Functional status	41.1 (14.1)	34.5 (14.5)	27.2 (16.4)	**12.37**	0.23	0.59	**11.60**	0.21
**Secondary outcomes**								
Self-efficacy	32.4 (10.8)	43.5 (10.0)	43.1 (12.0)	**−24.21**	0.53	1.06	0.66	0.001
*Dysfunctional behavioral cognitions*								
Catastrophizing	22.9 (8.8)	14.3 (7.0)	12.3 (7.5)	**23.73**	0.52	1.08	**6.68**	0.08
Fear of movement	39.6 (6.4)	32.7 (6.9)	31.8 (7.3)	**23.01**	0.50	1.06	**3.11**	0.02

Values in bold are statistically significant (*p* < 0.05)

*FU 1 yr* 1-year follow up; *Pre* pre-treatment assessment; *Post* post-treatment assessment

*t* t-value paired *t*-test; *d* Cohens’ d effect size

*R*^*2*^ treatment effect

#### Unique contribution of self-efficacy to post-treatment disability

After correction for the influence of pre-treatment values for functional disability, pain intensity, catastrophizing and fear of movement, the proposed multivariate linear basic model to predict post-treatment functional status based on post-treatment dysfunctional behavioural cognitions was found to be significant (*R*^2^ = 0.49, F_(6,517)_ = 83.67, *p* < 0.001; Table 3). Only pre-treatment pain intensity had no significant influence on the outcome (B = 0.22 [SE = 0.02], *t* = 1.06, *p* = 0.291). Addition of self-efficacy improved the basic model (final model: *R*^2^ = 0.57, F_(8,515)_ = 85.20, *p* < 0.001; Table 3), showing that after correcting for the pre-treatment value, only post-treatment self-efficacy significantly contributed (B = -0.56 [SE = 0.06], *t* = − 9.26, p < 0.001) to improved functional status, with no significant influence of post-treatment dysfunctional behavioural cognitions (catastrophizing B = 0.14 [SE = 0.09], *t* = 1.67, *p* = 0.095; fear of movement B = 0.08 [SE = 0.06], *t* = 1.05, *p* = 293). This was further substantiated by the relative contribution of post-treatment self-efficacy to post-treatment functional status, expressed in standardized betas, which is 5.67 times more than post-treatment.

**Table 3:** Predictive indicators of post-treatment functional status, adjusted for baseline functional status and pain intensity

	Basic model		Final model			
	B	SE B	B	SE B	β	95% CI
*Post-treatment*						
Catastrophizing	0.49	0.08	0.14	0.09	0.07	[− 0.25, 0.31]
Fear of movement	0.24	0.08	0.08	0.08	0.04	[−0.07,0.24]
Self-efficacy			−0.56	0.06	−0.39	[−0.68, − 0.44]
*Pre-treatment*						
Catastrophizing	−0.20	0.06	−0.13	0.06	−0.08	[−0.25, − 0.01]
Fear of movement	− 0.30	0.09	− 0.26	0.08	−1.12	[− 0.42,-0.11]
Self-efficacy			0.05	0.05	0.03	[−0.05, 0.16]
Functional status	0.63	0.04	0.51	0.04	0.50	[0.43, 0.58]
Pain intensity	0.02	0.02	0.02	0.02	0.03	[−0.02, 0.06]
Constant	8.87	3.19	43.84	5.15	–	[33.71, 53.96]
Model						
F	83.67		85.20			
*R*^2^	0.49		0.57			

Multivariate linear regression models

Values in bold are statistically significant (p < 0.05)

*B* unstandardized beta coefficient; *β* standardized beta coefficient

*SE* standard error; *CI* confidence interval

## Discussion

To understand the complexity of LBP the well-established cognitive-behavioural ‘fear avoidance model’ [29, 35] is used to explain the development of chronic LBP (CLBP) from an acute episode, and the maintenance of persistent pain leading to pain-related disability [2, 29, 36]. However, the working mechanisms of multidisciplinary bio-psychosocial pain management programs, based on cognitive behavioural training (CBT) principles, are still poorly understood. This study showed that targeting self-efficacy during an intensive two-week bio-psychosocial combined physical and psychological (CPP) pain management program is an important driver to a fast improvement of functional status in patients with longstanding and persistent chronic low back pain (CLBP). Participants in this program are able to learn and apply pain self-management principles in a short amount of time. They learn to cope with dysfunctional behavioural cognitions (pain catastrophizing and fear of movement) and, based upon a previously published study, they are able to manage their back problems at least five years [10].

### Improvement in dysfunctional behavioural cognitions and self-efficacy

In this study the improvements in catastrophizing, fear of movement, and self-efficacy observed at post-treatment, were maintained at one-year follow-up, and appeared larger compared to those reported in previous studies [37, 38]. However, the findings in these studies are based on less intensive programs compared to the highly intense program under study. Intense programs are assumed to reduce pain severity and to improve functionality in patients with CLBP more effectively than those that are less intensive. Although evidence to support this remains controversial [39, 40], it is tempting to suggest that the large effect sizes and treatment gains reported during those two weeks were the result of the intense program. Other non-specific factors (moderators), such as such a highly structured and standardized program, clear treatment rationale and treatment goals combined with a trained and highly skilled staff could also have attributed to this effect [38, 41–43]. Further research is needed to determine whether the treatment effect could be attributed to the ‘pressure cooker’ concept or to any of the other moderators.

### Key finding: unique contribution of self-efficacy to post-treatment disability

Although the relevance of targeting self-efficacy is shown in heterogeneous chronic pain samples [13], and the mediating role in CLBP is acknowledged in cross-sectional studies [16, 17, 44] evidence still lacks showing its contribution and role in the treatment of CLBP. The key finding of the current study is that self-efficacy is a more important and potent driver for fast improvements in functional outcome than catastrophizing and fear of movement. Adding changed (i.e. improved) self-efficacy significantly contributes to post-treatment functional outcome and also improved the basic model (Table 3, *R*^*2*^ = 0.49 [basic model] to *R*^*2*^ = 0.57 [final model]). The contribution of dysfunctional behavioural cognitions (catastrophizing and fear of movement) was not significant and considerably less than shown in the basic model (Table 3). The unique contribution of improved self-efficacy is also reflected in the standardized betas; almost 6 (catastrophizing) and 10 times (fear of movement) higher.

As catastrophizing, fear of movement, and self-efficacy significantly and relevantly improve during the two-week intensive program, and are maintained at least for a year, and acknowledging the fact that the constructs of these indicators at least partially overlap [2], this suggests that improvement of catastrophizing and fear of movement is achieved by improved self-efficacy. Furthermore, these rapid treatment gains can be achieved irrespective of the initial level of self-efficacy believes, catastrophizing, fear of movement, and experienced pain intensity. Notably, post-hoc analyses showed that improvements in dysfunctional behavioral cognitions and self-efficacy beliefs did not differ between patients with or without previous surgery (Pearson Chi^2^ ranging from 0.03 to 1.14 [*p* > 0.05]). The study results indicate that targeting self-efficacy during the program increases the patients’ believe that they are able to achieve improved functional outcome. As such, they seem to quickly learn and integrate self-management principles to control dysfunctional behavioural cognitions and achieve improved functional outcome. This supports the evidence that self-efficacy is a more important determinant than fear of movement in explaining disability [16, 17]. Besides a systematic review in heterogeneous chronic pain samples [13], to our knowledge this study is the first to show the relevance of supporting the patient’s self-efficacy to facilitate recovery in terms of lower levels of disability in large sample of selected patients with CLBP.

It is tempting to suggest that targeting self-efficacy facilitates recovery in patients with longstanding CLBP. Despite the fact that the explained variance of the final model was relatively high (*R*^*2*^ = 0.57, Table 3), other (non-specific) moderating factors, as afore mentioned, might also be of influence in post-treatment functional outcome. In fact, during the two-week program a wide range of CBT and exposure techniques are used, sustaining the educational part of the program, which are aimed at changing both self-efficacy and dysfunctional behavioural cognitions in order to achieve improved functional outcome. These findings seem to imply that education at least to some extent, could be responsible for the resulting strong effect in the primary functional outcome that was found. Furthermore, a multidisciplinary team of allied healthcare professionals (e.g. psychologist, physiotherapist, psychomotor therapist) usually leads these programs. This suggests that supervision by a medical specialist might not be instrumental for treatment success, though this remains to be confirmed.

### Strengths and limitations

An important strength of this study is the large sample size (*n* = 524) and the low percentage of missing data. Although 67 patients (13%) had incomplete data for at least one assessment, no pre-treatment differences were seen with those with complete data. By using imputation techniques data of all patients could be used and as such the conclusions based on the study findings are robust.

Some limitations of this study need to be mentioned. First, due to the selection procedure for the program generalization to common practice might be limited, as only motivated patients with a high willingness to change their behaviour were included. Patients vary on the extent to which they are willing and ready to adopt a self-management approach to their problem. Highly motivated patients, who believe that a pain management approach would help or who have decided to adopt a self-management approach, are assumed ready to change their pain-related behaviour. As such, motivation or willingness to change might be of importance for treatment dosage [42] and as selection criterion to match treatments [41]. Second, the fear avoidance model suggests a sequential progression for dysfunctional behavioural cognitions. Although our study confirmed that changes in dysfunctional behavioural cognitions occurred during the two-week program, it can neither clarify the exact moment during the program at which these changes occurred, nor it was the focus of the current study. It is also necessary to consider that the sequential parameters of relations among catastrophizing, fear of movement and disability may differ for the development of CLBP in contrast to recovery of disability as a result of an intervention [45].

In conclusion, in the treatment of selected and motivated patients with longstanding persistent CLBP, targeting self-efficacy has a unique contribution to a fast improvement of functional status. In treatments for CLBP as CPP pain management programs and (online) self-management programs targeting patients’ self-efficacy should have a prominent place.

## Data Availability

The data used and/or analysed during the current study are available in the text and from the corresponding author on reasonable request.

## Abbreviations

CBT: Cognitive behavioural therapy
CLBP: Chronic low back pain
CPP program: Combined physical and psychological program
FoM: Fear of movement
LBP: Low back pain
MCIC: Minimal clinical important change
NRS: Numeric rating scale
ODI v2.1a: Oswestry disability index version 2.1a
PCS: Pain catastrophizing scale
PSEQ: Pain self-efficacy scale
STROBE: Strengthening the reporting of observational studies in epidemiology
TSK: Tampa scale for kinesiophobia
